# Automatic plane adjustment of orthopedic intraoperative flat panel detector CT-volumes

**DOI:** 10.1117/1.JMI.9.3.034001

**Published:** 2022-05-09

**Authors:** Celia Martín Vicario, Florian Kordon, Felix Denzinger, Jan Siad El Barbari, Maxim Privalov, Jochen Franke, Sarina Thomas, Lisa Kausch, Andreas Maier, Holger Kunze

**Affiliations:** a Friedrich-Alexander-Universität Erlangen-Nürnberg, Pattern Recognition Lab, Erlangen, Germany; b Friedrich-Alexander-Universität Erlangen-Nürnberg, Erlangen Graduate School in Advanced Optical Technologies, Erlangen, Germany; c Siemens Healthcare GmbH, Forchheim, Germany; d BG Trauma Center Ludwigshafen, Department for Trauma and Orthopaedic Surgery, Ludwigshafen, Germany; e German Cancer Research Center, Division of Medical Image Computing, Heidelberg, Germany

**Keywords:** multiplanar reconstruction, orthopedics, flat panel CT, plane regression

## Abstract

**Purpose:**

To assess the result in orthopedic trauma surgery, usually three-dimensional volume data of the treated region is acquired. With mobile C-arm systems, these acquisitions can be performed intraoperatively, reducing the number of required revision surgeries. However, the acquired volumes are typically not aligned to the anatomical regions. Thus, the multiplanar reconstructed (MPR) planes need to be adjusted manually during the review of the volume. To speed up and ease the workflow, an automatic parameterization of these planes is needed.

**Approach:**

We present a detailed study of multitask learning (MTL) regression networks to estimate the parameters of the MPR planes. First, various mathematical descriptions for rotation, including Euler angle, quaternion, and matrix representation, are revised. Then, two different MTL network architectures based on the PoseNet are compared with a single task learning network.

**Results:**

Using a matrix description rather than the Euler angle description, the accuracy of the regressed normals improves from 7.7 deg to 7.3 deg in the mean value for single anatomies. The multihead approach improves the regression of the plane position from 7.4 to 6.1 mm, whereas the orientation does not benefit from this approach. Thus, the achieved accuracy meets the reported interrater variance in similarly complex body regions of up to 6.3 deg for the normals and up to 9.3 mm for the plane position.

**Conclusions:**

The use of a multihead approach with shared features leads to more accurate plane regression compared with the use of individual networks for each task. It also improves the angle estimation for the ankle region. The reported results are in the same range as manual plane adjustments. The use of a combined network with shared parameters requires less memory, which is a great benefit for the implementation of an application for the surgical environment.

## 1 Introduction

The default imaging modality to assess fracture reduction, implant position, and overall outcome during an orthopedic trauma surgery is x-ray imaging. However, the success of the surgery cannot be clearly judged solely from the x-ray image in complex anatomical regions such as calcaneus, ankle, wrist, or knee. Due to overlapping or convex bones, assessing the positions of implants with respect to the corresponding bones is difficult. Therefore, the acquisition of three-dimensional (3D) scans is recommended before releasing the patient from the hospital. If 3D imaging is performed postoperatively, e.g., using a diagnostic computed tomography (CT) system, not every minor finding will lead to revision surgery, which will spare the patient the risks of additional surgery. However, recent studies have shown that intraoperative 3D imaging has led to corrections for up to 40% of surgeries, depending on the body region.^1^^–^^10^ Thus intraoperative 3D imaging reduces the number of revision surgeries and improves the outcome of surgeries because minor findings are also usually corrected.

For intraoperative acquisition of 3D volumes, mobile C-arm systems, which are capable of cone-beam tomography (CBCT), are usually employed. These systems typically have a relatively limited field of view with a volume edge length of about 160 to 250 mm. Consequently, the captured anatomy section and thus the anatomical landmarks’ position and visibility may vary substantially.

When reading a 3D volume, the volume should be aligned to the anatomical structures in a standardized way as it is done in the radiology department. The key slices that contain anatomical structures that are decisive for assessing intervention results are called standard planes. Typically there are three of them: the axial, coronal, and sagittal planes. From an intraoperative 3D volume, they are typically obtained by the multiplanar reconstruction (MPR) technique. Generally, the three planes are orthogonal to each other, but in some regions, instead of these three orthogonal planes, an oblique plane provides the required information. One example of an oblique plane is the semicoronal plane in the calcaneus region, a variation of the coronal plane that is not orthogonal to the axial and sagittal planes and which allows for the evaluation of the reconstruction of the posterior talar surface.^11^

In Ref. 12, it was shown that the accuracy of surgeons adjusting the standard MPRs highly depends on the region. In the lumbar spine region, where the planes can be adjusted using well-defined landmarks, the interrater difference was about half compared with the proximal femur region, where these kinds of landmarks are missing. The mean interrater variance was measured up to 6.3 deg for the normals and up to 9.3 mm for the plane position.

As mobile C-arms systems lack information about the spatial relationship between the system and the anatomical region, the adjustment of the plane position and orientation needs to be performed at the workstation in the operating room. This alignment of the planes is a manual task that takes 46 to 210 s depending on the experience level of the surgeon and, thus, is a time-consuming step in a surgery.^13^^,^^14^

Slice alignment in acquired volumes is a rather old topic. Although the initial focus was on automatic rotation of the brain CT,^15^^–^^17^ with the invention of 3D capable mobile C-arms systems—which were used mainly in orthopedic and trauma surgery environments—other body parts such as extremities attracted increased attention from researchers. Speeded up robust features were used by Brehler et al.^14^ to register the acquired volume with an atlas that has annotated MPR planes. This method requires the careful choice of the atlas and feature extraction method, but even then, this approach has a limited capture range of rotation. Therefore, in Ref. 18 shape models with attached labels for the MPR planes were used. For generating the shape models, multiple volumes need to be manually segmented, which is time-consuming. To account for small volume sizes that lead to cropped bones, and to be invariant to different metal implants positions, much effort and domain knowledge during the registration was applied to obtain a robust algorithm for one region. This leads to a long execution time of 23 s for the shape model registration and the subsequent plane regression.

Artificial intelligence systems allow for performing this task in a considerably faster time. An active research field for standard plane regression task is ultrasound imaging, for which in Ref. 19 probabilistic boosting trees were used to estimate nine transform parameters of the target MPRs using a multistage approach. Although it has a complex algorithmic design, this method achieves an average rotation error of 11.3  deg±8.0  deg, which does not meet clinical requirements. Li et al.^20^ proposed an iterative approach in which a CNN repeatedly estimates the transform between a two-dimensional (2D) plane and the standard plane. Using this approach, they circumvent a fully 3D approach as only a small number of plane samples and updates are necessary until the regression converges. The method, which predicts the transformation parameters for one plane at a time, achieves a rotation error of 12.7 deg and 12.6 deg for the transventricular and transcerebellar planes, respectively. Similar to the approach of Lu et al., this inference error substantially exceeds the reported interrater variance. Especially for more complex anatomies in which the 3D spatial information cannot be captured well on 2D projections and more large-scale structures that spread the entire field of view are of interest, a 3D-based algorithm could be beneficial.

In a more general domain, spatial transformer networks (STN)^21^ predict the parameters of an affine transform matrix that is used to spatially manipulate feature maps in a convolutional architecture. No direct supervision for the transform is used, allowing the network to optimize toward a spatial configuration that maximizes the performance of the actual supervised target task. The Ω-net by Vigneault et al.^22^ modifies this approach by estimating the transform parameters for direct manipulation of the input image data. Based on the feature maps of a prior segmentation CNN, direct ground truth for the transformation parameters is used to bring the input images to a canonical form that better suits the downstream segmentation task. Despite the reported rotational transformation error being promising (95% of rotation errors within ±0.63  rad), the additional segmentation module imposes computational overhead, which is unwanted in a surgical setting where expected execution speed and hardware limitations favor rather lightweight algorithms.

Martín Vicario et al.^23^ used a PoseNet for the regression of the plane parameters. These plane parameters can be interpreted as transformation parameters. Comparing the structure of the PoseNet with that of the STN, it can be clearly seen that the convolutional layers resemble the localization network and the fully connected layers resemble the final regression layer. Thus, Martín Vicario et al.^23^ avoided the additional overhead of the segmentation introduced by the Ω-net while retaining the approach of supervising the transform parameters, which are of interest for the current task.

In Ref. 23, separate networks were used for different anatomical regions. For each region, a single network for the regression of all three plane parameters achieved the best performance. However, they did not analyzed how one single network performed for all body regions.

This article contributes in multiple ways:

- We extend our initial ablation study presented in Ref. 23 by a comparison of four different MPR plane parameter representations, including an additional rotation representation and comparing it with the previously published results. We also increased the number of evaluated body regions by adding proximal tibia (knee) and distal radius (wrist) to the calcaneus and ankle.
- We add a study of the single-task approach performance of dependency on the number of volumes, analyzing the generalization problem given the number of available data.
- We analyze different multitask learning (MTL) approaches to improve the performance of the baseline algorithm. Typically, the number of available volumes per body region is small. Caruana^24^ showed that MTL can help to find the right shared representation for related tasks when only a little data is available for the single tasks. Therefore, simultaneous learning for several tasks can help to find more appropriate representations and thus reduce the risk of overfitting. Furthermore, such combined training of MPR regression for different body regions can help to improve regression performance. We want to make use of this property of MTL in this work.

The approach of MTL also has a practical benefit: the MPRs are adjusted after the reconstructed volume was loaded into the volume viewer, and the body region of the volume is classified. Then, by having the body region class derived, a single task network is chosen and loaded from a hard drive to a graphics card. Measurements show that the parameter loading takes up to 1 s. A combined network, which can be used for the regression of MPRs in several body regions, is loaded once and then stays in memory, which is beneficial as it decreases the waiting time for the surgeon. Therefore, we compare two strategies with the results of region-specific networks. Both MTL strategies utilize a common encoder structure. The first approach uses a single head consisting of two fully connected layers for the different body regions, and the second approach—a multihead approach—implements separate heads for the individual body regions.

In Sec. 2, we present the employed mathematical description of planes. We describe the normalization of the coordinate system and introduce the different neural network architectures that we want to compare. Furthermore, the cost function for optimization is introduced. The implementation and the data that we use for training and testing, as well as the study design, are described in Sec. 3. After that, we present and discuss the results of our experiments in Sec. 4.

## 2 Methods

### 2.1 Plane Description

In this section, we recapitulate what MPRs are and how an MPR plane are described. MPRs are plane intersections of a volume. An MPR plane is described by its center position A and the linearly independent unit vectors $e_{u}$ and $e_{v}$ showing in the directions of the rows and columns. Each point on the plane fulfills the following equation:

$$P_{\lambda ,\mu }=A+\lambda e_{u}+\mu e_{v}.$$ (1)

The plane normal $e_{w}$ is the cross-product of these two direction vectors. Thus, the MPR plane can be associated with a translation defined by A and a rotation defined by $e_{w}$. These two transformations define which structures the plane displays. The choice of $e_{u}$ and $e_{v}$ being orthogonal to $e_{w}$ defines the in-plane rotation of the displayed content. In the case of three orthogonal standard MPRs, the MPR planes may share the same orientation vectors of course with different meanings. For the semicoronal plane, however, $e_{v}$ and thus $e_{w}$ are different from the orientation vectors of the axial and sagittal MPR plane. To obtain a generic framework, we opt for a separate regression of the MPR rotation. Additionally, we regress the center position of the plane and not the intersection point; thus, A is different for all planes.

Based on the parametrical description, we derive three additional ways to describe the pose of the plane. Previous studies^25^^,^^26^ have shown that the rotation representation can impact the quality of the algorithm. As we show, dependent on the representation, loss functions that are connected more closely to the error can be selected.

Rewriting Eq. (1), the point $P_{\lambda ,\mu }$ is also described as

$$P_{\lambda ,\mu }=[\begin{array}{cccc} e_{u} & e_{v} & e_{w} & A\\ 0 & 0 & 0 & 1 \end{array}][\begin{array}{c} \lambda \\ \mu \\ 0\\ 1 \end{array}].$$ (2)

So the plane can be associated with an homogeneous transformation T from the plane coordinate system to the volume coordinate system, which consists of a 3×3 rotation matrix $R=[e_{u}e_{v}e_{w}]$ and a three-element translation vector t=A

$$T=[\begin{array}{cc} R & t\\ 0 & 1 \end{array}].$$ (3)

By the construction of R, its nine parameters are highly coupled. So, the column vectors are normalized, the dot product of two vectors is zero, and one column vector is calculated by the cross product of the other two vectors. These properties are utilized by the 6D method.^26^ With this method, the values of two vectors are estimated by the neural network. Typically, the first two columns are utilized. However, it might also be favorable to regress the first and the third column instead of the second column as it encodes the normal of the plane, which itself is part of the score function [Eq. (8)], which will be introduced below. We denote the 6D method, which regresses the parameters for the x and y directions with $6D_{xy}$, and the one that regresses the x and z directions with $6D_{xz}$. After regression of the values, each column vector is normalized, and the missing column vector is calculated as the cross product. As the matrix is a pure rotation matrix, its entries are in the range of [−1,1].

A more common way to regress rotation parameters is to decompose the matrix into Euler angles or use a unit quaternion representation. Euler angles suffer from discontinuous values, whereas the quaternion representation does not have this problem. To overcome the limitation for Euler angles, we follow Baltruschat et al.^25^ and Kausch et al.^12^ and do not directly regress the angular value but regress their sine and cosine values. The actual angle value is then calculated from the regressed values using the atan 2 method. Another advantage of this method is that the parameter range of the values is compressed into the range [−1,1]. The same range applies to the values of the quaternions.

The translation is normalized with respect to the volumes’ dimensions and thus also lies in the range of [−1,1] with the origin placed at the center of the volume.

### 2.2 Separate and Combined Networks

Because in Ref. 23 the regression of the MPR plane parameters of only two body regions was studied, in the experiments for this work, four body regions were included. Thus, the question of how one single network performs for all body regions arises.

**Fig. 1:**
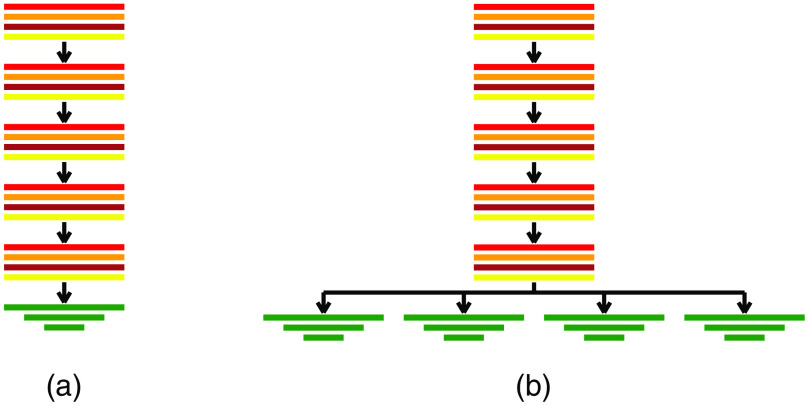
Schematic visualization of the analyzed network architectures. (a) Single-head network without providing body region information to the network. The five convolutional blocks consist of a 3D convolutional layer (red), followed by a ReLU activation function (orange), batch normalization (brown), and a max-pooling operation (yellow). The obtained features are fed into three fully connected layers (green). (b) Multihead network with convolutional blocks shared across body regions and individual fully connected layers for the different body regions.

In preliminary experiments, we compared the performance of the VGG-16,^27^ ResNet-34,^28^ and PoseNet^29^ networks. We observed that the PoseNet generalized better and was more robust compared with the other two architectures. Therefore, we chose the PoseNet as the baseline network for our study [Fig. 1(a)].

The PoseNet consists of five convolutional layers and three fully connected layers. The last layer has as many output nodes as regressed values. The topology of this baseline network is listed in Table 1. When we use this network for regression of the plane parameters, it is agnostic about the body region for which the planes’ parameters need to be calculated.

**Table 1:** Structure and parameter layout of the baseline network.

Block	Input resolution	# Input channels	# Output channels
CNN1	72×72×72	1	8
CNN2	31×37×31	8	16
CNN3	16×19×16	16	32
CNN4	8×10×8	32	64
CNN5	4×5×4	64	228
FC1	1×1×1	10,240	1300
FC2	1×1×1	1300	50
FC3	1×1×1	50	# parameters

As in Ref. 23, this information was provided by selecting the correct individual network. We want to compare the performance of this base network with a multihead approach with a shared convolutional feature extraction but individual fully connected regression heads for each anatomical region [Fig. 1(b)].^30^ During inference, the knowledge about the body region is used to select the head and output nodes that correspond to the given body region. During backpropagation, the error gradients for all other body regions are set to zero. Thus only parameters within the fully connected layers belonging to the selected body region and those within the convolutional layers are updated.

### 2.3 Augmentation and Value Normalization

During training, online augmentation of the volumes is employed. The spatial augmentation includes random rotation within the interval [−45,45]  deg, random spatial scaling of the volume by a factor in the range [0.95, 1.05], translation by [−12,12]  mm, center cropping, and subsampling. All of the aforementioned augmentations were applied with a probability of 0.5 and were sampled uniformly from the respectively given range. Additionally, mirroring in the x direction is added with a probability of 0.5, which allows for simulating left-right handedness of the volume. These spatial operations are composed by combining their representation by homogeneous matrices into a single composite matrix. The homogeneous transform matrix is given as

$$T_{m}=T_{r}T_{s}T_{t}T_{R},$$ (4)

where $T_{r}$, $T_{s}$, $T_{t}$, and $T_{R}$ represent the subsampling, scaling, translate, and rotation homogeneous matrices, respectively. This implementation helps to speed up the calculation and reduces the number of performed interpolations to one.

Thereafter, an intensity augmentation is implemented to simulate that the hounsfield unit (HU) values of mobile C-arm devices are generally not as well calibrated as those of CT systems. Thereto, the value of 1000 HU is added to the interpolated HU values, and the result is multiplied by a factor uniformly sampled from the range [0.95, 1.05]. For normalization, the approach of Martín Vicario et al.^31^ was implemented: a windowing function w(x) is applied after clipping the volume intensity values to the range of [−490,1040]  HU and rescaling it to [0, 1]. The resulting intensity value before applying the windowing function is given as

$$c(x)=\{\begin{array}{ll} 0 & \text{if}\;x<\min,\\ \frac{f(x+1000)-\min}{\max-\min} & \text{if}\;\min<x<\max,\\ 1 & \text{if}\;x>\max. \end{array}$$ (5)

where f represents the random factor. The windowing function is defined as

$$w(x)=\frac{1}{\left(1+e^{g(0.5-x)}\right)},$$ (6)

with a minimum and maximum values dependent gain factor. The gain factor is given as

$$g=\log(\frac{1-y}{y})/0.4,$$ (7)

where y=0.02(max−min). In contrast to min–max normalization, it reduces the signal variance of metal and air, which typically contains little to no information about the plane’s parameters.

### 2.4 Postprocessing of Regressed Values

In Ref. 23, it was shown that a combined regression of the parameters of the three planes is beneficial compared with training separate networks for each plane. So the accuracy can be improved when the planes are redundantly regressed. In the same publication, it was also shown that the training does not benefit from an additional orthogonality constraint on the regressed values. Therefore, we decided to regress the parameters of the planes in all of the presented architectures decoupled and adjust them afterward algorithmically.

As presented in Ref. 23, the axial plane is the most accurately regressed in the anatomical regions. Therefore, it is taken as reference plane for the other planes. This means that the in-plane rotation of the coronal and the sagittal plane is corrected such that the intersection of the axial plane at these planes is at 0 deg. Thereafter, in cases in which the planes are orthogonal to each other, the normal direction of the sagittal plane is adjusted to be orthogonal to the axial and coronal planes.

It can be shown that this kind of postprocessing helps to improve the accuracy of the normal’s angle and inplane rotation by up to 1.89 deg. For more details, see Appendix A.

## 3 Experiments

### 3.1 Data Sets

**Fig. 2:**
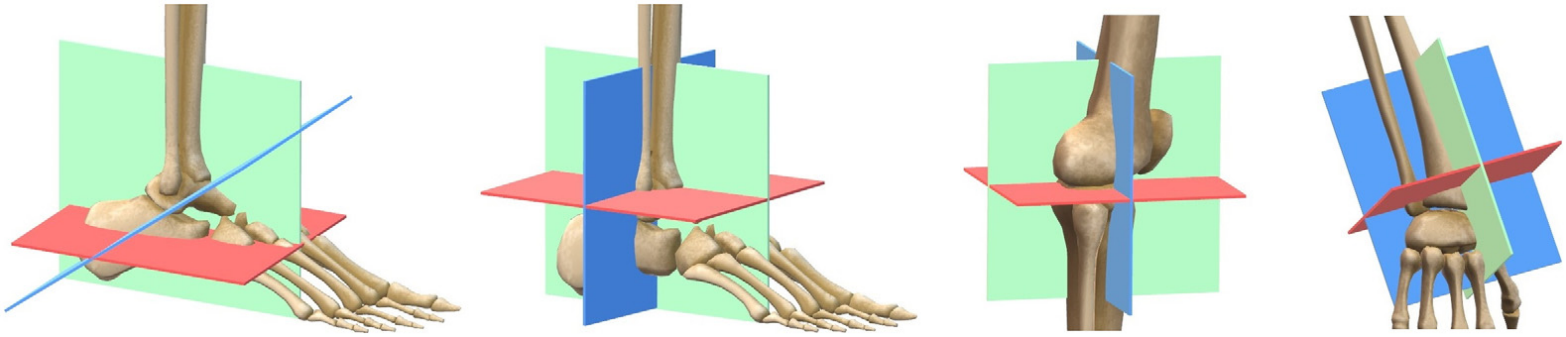
Representation of the 3D definition of axial (red), (semi-)coronal (blue), and sagittal (green) standard planes in the calcaneus, ankle, knee, and wrist.

Our data set consists of 160 volumes of the calcaneus region, 220 volumes of the ankle region, 274 volumes of the knee, and 250 volumes of the wrist. All volumes were acquired with a mobile C-arm system Cios Spin from Siemens Healthineers and reconstructed offline with the Feldkamp–David–Kress algorithm using parameters equal to the product standard settings. The volumes have a uniform resolution of $512^{3}\;\;\text{voxels}$ and a field of view of ${\left(160\;\mathrm{mm}\right)}^{3}$. They were partly acquired after an orthopedic surgery for assessing the surgical result and partly from cadavers that were prepared for surgical training. The cadaver data sets were typically scanned twice: once without any metal and once with metal objects put on the surface of the cadaver. We also obtained volumes of cadavers with various metal implants acquired during surgical training. The exact distribution of the data sets is listed in Table 2. All available volumes were included in the data set, without any constraint on the positioning of the body part of interest. The volumes were corrected for incorrect patient position description according to the digital imaging and communications in medicine (DICOM) image meta information. For each body region five data splits were created, taking care that volumes of the same patient belonged to the same subset and that the distribution of the data set’s origin was approximately the same as in the total data set. For all volumes, standard planes were defined according to the clinical definition provided in Ref. 11. Sketches of the planes are displayed in Fig. 2.

**Table 2:** Number, origin, and realism with respect to metallic objects of the volumes.

	Cadaver			Clinical	Total
	Metal implants	Metal outside	No metal	Metal implants	
Calcaneus	9	63	62	26	160
Ankle	36	61	56	67	220
Knee	65	68	70	71	274
Wrist	0	101	102	46	249

For the ankle, knee, and wrist volumes, axial, coronal, and sagittal MPRs were annotated, and for the calcaneus data sets, axial, sagittal, and semicoronal planes were annotated. This was done by a medical engineer after 5 h of training using a syngo XWorkplace VD20 that was modified to store the plane description. Axial, sagittal, and coronal MPRs were adjusted with coupled MPRs. The semicoronal plane was adjusted thereafter with decoupled planes. The annotation validity was verified by an expert physician and additionally by a senior medical engineer.

### 3.2 Performance Metric

As an evaluation metric to compare the performance of the networks, we use a weighted average over the individual error values of the three regressed planes:

$$p=\frac{1}{\#\text{planes}}\underset{j\in \text{planes}}{\sum }0.2d_{j}+0.6\epsilon_{n,j}+0.2\epsilon_{i,j},$$ (8)

where $d_{j}$ denotes the mean error of the absolute translation of the center in the direction of the j’th plane’s normal. $\epsilon_{n,j}$ is the deviation of the normal vectors $e_{w}$, and $\epsilon_{i,j}$ is the in-plane rotation error calculated as the mean difference angle of $e_{u}$ and $e_{v}$, after projecting the directions on the plane defined by the annotation. The different weights in Eq. (8) were chosen heuristically and reflect that the normal has the most complex effect on the result. For this normal to be corrected, out-of-plane rotations would be necessary, whereas in-plane rotation and plane translation are easy-to-fix components.

In the results tables below, the mean and standard deviation of the median prediction errors of the folds are represented.

### 3.3 Study Design

Before investigating a combined regression network for multiple anatomies, some further experiments were carried out to evaluate the performance of the baseline network. We have seen in Sec. 2 that there are several possibilities for parameterizing rotations. In addition to Martín Vicario et al.,^23^ the $6D_{xz}$ method was introduced, taking into account that the main contribution to the performance metric comes from angular deviation of the normals. Therefore, as the first experiment, a comparison of the representation with Euler angles, quaternions, $6D_{xy}$, and $6D_{xz}$ is performed for the four body regions.

The best performing representation is used in the subsequent experiments.

In Ref. 23, the question of whether better results can be expected with more data samples was kept open. Because the number of available volumes is fixed, we incrementally reduce the number of volumes used for training. For this, the training for the different body regions is repeated using 100%, 80%, 60%, and 40% of the volumes in the training split, while keeping the test volumes unchanged.

Following the evaluation of the baseline model, different experiments were carried out to evaluate the performance of using a single model for all body regions. First, we trained a single network for all body regions without providing any further class information. Second, a multihead architecture [Fig. 1(b)] is used: all body regions share the convolutional feature extraction layers but are individually processed in separate regression heads consisting on three fully connected layers for each anatomical region. To overcome the imbalance between the different classes, the volumes were randomly over-sampled from the minority classes with a weight given by the number of volumes from a given class.

### 3.4 Implementation

The models are implemented in PyTorch (v.1.5.1) and trained on Windows 10 systems with 32 GB RAM and 8 GB NVIDIA RTX 2070S. The weights are initialized by the He et al. method.^32^ The network is trained by a minibatch gradient descent optimizer with momentum. For optimization of the network parameters, the mean squared error between model prediction and ground truth was calculated at each output node. The total number of epochs was set to 400, verifying training convergence of all model variants. For the selection of the learning rate, learning rate decay, step size, momentum, and batch size, a hyperparameter optimization was performed (for details of hyperparameter optimization see Appendix B).

## 4 Results

As can be observed in Table 3, the evaluation of the different rotation representations in the base model shows that the 6D method outperforms the Euler and quaternion representations in all body regions except the knee. For this region, similar performance to the best representation, the Euler angles, is reached. Among the 6D methods, no (noticeable) difference in performance between $6D_{xz}$ and $6D_{xy}$ can be observed. Thus, using the normal in the directly obtained values and consequently also in the cost function does not generally improve the quality of the planes parameter regression. In two-body regions, we observed a small reduction in the mean error of the estimated normals, whereas an error increase was registered for the other two regions. In all cases, the in-plane rotation performance got significantly worse. The position estimation error of the planes was approximately the same for both representations. Due to these reasons, the $6D_{xy}$ variant was chosen for the remaining experiments. The use of sine and cosine representations of the Euler angles instead of the raw angle values shows superior performance over the quaternion representation for the estimation of the plane normal. Looking at the performance score that weights all metrics (Sec. 3.2), the Euler angles show better results in three body regions compared with the quaternions.

**Table 3:** Summarized results of evaluation of Euler angles, quaternions, $6D_{xy}$, and $6D_{xz}$ rotation representations in standard plane regression of calcaneus, upper ankle, knee, and wrist regions.

	d (mm)	$\varepsilon_{n}$ (deg)	$\varepsilon_{i}$ (deg)	Score
Calcaneus				
Euler	14.39 ± 1.64	8.93 ± 1.60	9.99 ± 0.75	10.23 ± 1.11
Quat.	9.93 ± 2.53	9.96 ± 1.75	9.57 ± 1.52	9.87 ± 1.65
$6D_{xy}$	9.94 ± 1.92	**8.08 ± 0.38**	**8.09 ± 0.45**	**8.46 ± 0.63**
$6D_{xz}$	**9.31 ± 1.10**	8.23 ± 0.69	9.42 ± 1.02	8.68 ± 0.65
Ankle				
Euler	7.78 ± 0.36	6.98 ± 0.77	7.52 ± 0.76	7.25 ± 0.66
Quat.	**5.00 ± 0.09**	8.16 ± 0.79	8.31 ± 0.71	7.56 ± 0.63
$6D_{xy}$	5.43 ± 0.25	6.61 ± 0.34	**6.37 ± 0.31**	6.32 ± 0.25
$6D_{xz}$	5.41 ± 0.49	**6.17 ± 0.78**	7.32 ± 1.06	**6.25 ± 0.65**
Knee				
Euler	**6.81 ± 0.65**	**6.59 ± 1.05**	7.36 ± 1.54	**6.79 ± 0.96**
Quat.	6.82 ± 0.72	9.45 ± 0.67	10.54 ± 1.22	9.15 ± 0.59
$6D_{xy}$	**6.81 ± 0.47**	6.71 ± 0.63	**7.07 ± 0.95**	6.80 ± 0.55
$6D_{xz}$	7.15 ± 1.16	7.19 ± 0.52	8.22 ± 0.62	7.39 ± 0.49
Wrist				
Euler	7.45 ± 1.00	8.35 ± 1.66	9.82 ± 1.38	8.48 ± 1.31
Quat.	8.46 ± 1.93	11.31 ± 1.87	13.47 ± 2.48	11.22 ± 1.83
$6D_{xy}$	7.27 ± 1.08	7.74 ± 1.14	**8.72 ± 0.64**	7.85 ± 0.94
$6D_{xz}$	**7.21 ± 1.02**	**7.21 ± 1.06**	9.37 ± 1.08	**7.64 ± 0.84**

Note: Bold values represent the lowest values of error class for each body region.

**Fig. 3:**
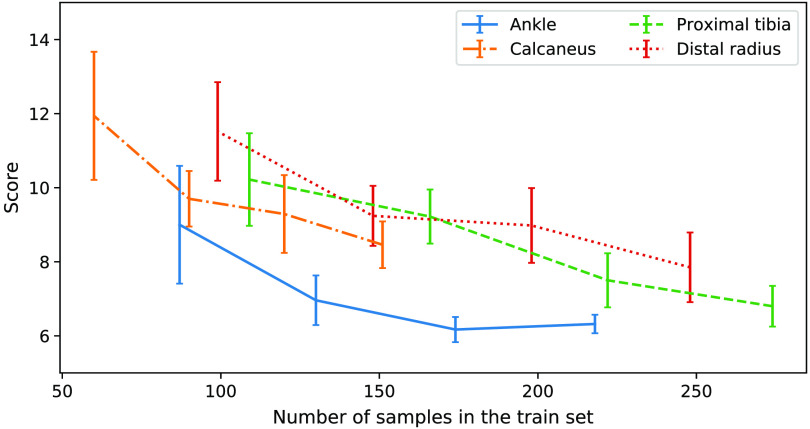
Evolution of the performance score upon reduction of the number of samples used in the training split in calcaneus, ankle, knee, and wrist body regions. From left to right, the number of volumes for evaluation of each body region correspond to the results of training with 40%, 60%, 80%, and 100% of the total number of samples in the train set.

The performance analysis of the baseline model upon reducing the amounts of training data (Fig. 3) reveals that, in the ankle body region, 174 volumes are sufficient for finding good results. For the other body regions, the number of provided volumes should be increased to obtain the best possible results. Compared with the ankle, the other regions show a larger variance in shape and joint angulation, and thus more training data is needed to capture all different shapes. It can be observed that calcaneus, knee, and wrist regions all show similar performance characteristics at reduced amounts of training data.

The comparison of the multihead networks (Table 4) shows that a combined network that jointly estimates the parameters of the planes for different body regions can improve the accuracy of the planes positions. For calcaneus, ankle, and knee, the improvement is substantial. However, for the angle regression task, this network variant yields inferior results for calcaneus and wrist. As the angular errors have a higher impact on the score, the overall performance is inferior for these two body regions. The multihead network proves to be significantly better for the ankle and knee regions than individual models with a p-value, in both cases, that is lower than 0.001. For the calcaneus region, the single-task network and the multihead network have about the same performance, with their mean performance score and rotation errors lying in each others range of standard deviation. The p-value shows that the difference between these two methods is not significant. Only for the wrist body region are the angle errors, and thus also the score, significantly worse compared with the single task network. For this region, the multihead network achieved the worst values compared with all MTL network variants.

**Table 4:** Summarized results of the different networks including the use of single-task models (a model for each anatomy), the model for training all anatomies using a single head, and the multihead model. The p-value is obtained by applying a paired t-test between the score results of single-task models and the singlehead and multihead models.

	d (mm)	$\varepsilon_{n}$ (deg)	$\varepsilon_{i}$ (deg)	Score	P-value
Calcaneus					
Single-task	9.94 ± 1.92	**8.08 ± 0.38**	**8.09 ± 0.45**	**8.46 ± 0.63**	—
Single-head	9.17 ± 0.64	9.18 ± 1.21	8.87 ± 1.34	9.12 ± 1.03	<0.001
Multihead	**7.44 ± 0.31**	9.16 ± 1.80	8.55 ± 0.88	8.69 ± 1.23	0.037
Ankle					
Single-task	5.43 ± 0.25	6.61 ± 0.34	**6.37 ± 0.31**	6.32 ± 0.25	—
Single-head	6.34 ± 0.77	9.71 ± 1.98	9.64 ± 1.90	9.02 ± 1.59	<0.001
Multihead	**4.47 ± 0.33**	**6.08 ± 0.45**	6.61 ± 0.65	**5.86 ± 0.40**	<0.001
Knee					
Single-task	6.81 ± 0.47	6.71 ± 0.63	7.07 ± 0.95	6.80 ± 0.55	—
Single-head	6.71 ± 0.72	8.04 ± 0.58	8.14 ± 1.10	7.79 ± 0.53	<0.001
Multihead	**5.62 ± 0.68**	**6.70 ± 1.28**	**6.77 ± 0.81**	**6.49 ± 1.05**	<0.001
Wrist					
Single-task	7.27 ± 1.08	**7.74 ± 1.14**	**8.72 ± 0.64**	**7.85 ± 0.94**	—
Single-head	**6.42 ± 0.75**	10.34 ± 2.82	10.52 ± 2.04	9.59 ± 2.15	<0.001
Multihead	7.03 ± 1.16	10.50 ± 1.73	11.15 ± 1.29	9.93 ± 1.41	<0.001

Note: Bold values represent the lowest values of error class for each body region.

**Fig. 4:**
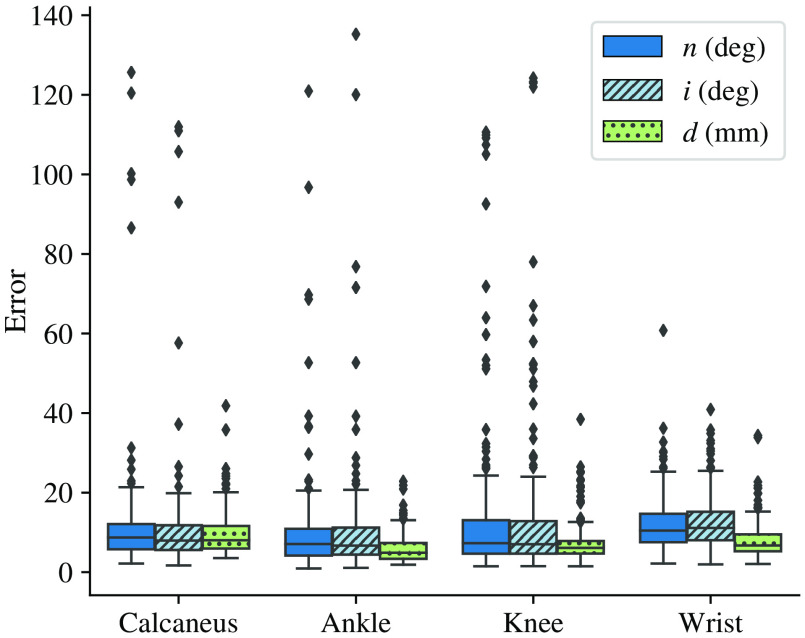
Individual distribution of plane and distance errors per anatomy obtained by the multihead network.

Across all experiments we could see that the estimation of the position can be improved by the MTL approaches (Table 4). However, the angle estimation for both the normals and the in-plane rotation do not benefit from the MTL approach (Fig. 4).

For a better understanding of this result, we compared the volumes contributing to the 10% best scoring results with those contributing to the 10% worst scoring results. The presence of metallic objects such as screws or plates were not observed as a source for these errors. We also discarded the possibility that the regression error was higher in the volumes in which only part of the relevant anatomy is shown. For these problematic cases, the algorithm is quite robust. However, in these volumes, we realized that the patient positioning was done in a different way in comparison with the standard, e.g., prone or left instead of supine or focus on the proximal femur instead of the tibial head. The high variance in positioning of the knee joint results in a substantially larger amount of outliers with performance scores >20. In comparison, the amount of outliers is decreased for the wrist joint, where standardized positioning is easier to achieve due to the small size and flexible configuration of the connecting anatomy. Thus, if the surgical setting permits it, it is recommended that the anatomy and the patient are positioned as uniform and standardized as possible, so the number of high-error observations is reduced.

Because the employed flip and rotation augmentation did not fully cover this variance in pose, additional training data needs to be added to handle this. The outliers in Fig. 4 are observed for body regions that were placed in clinically irrelevant positions and can be associated with this constraint.

**Fig. 5:**
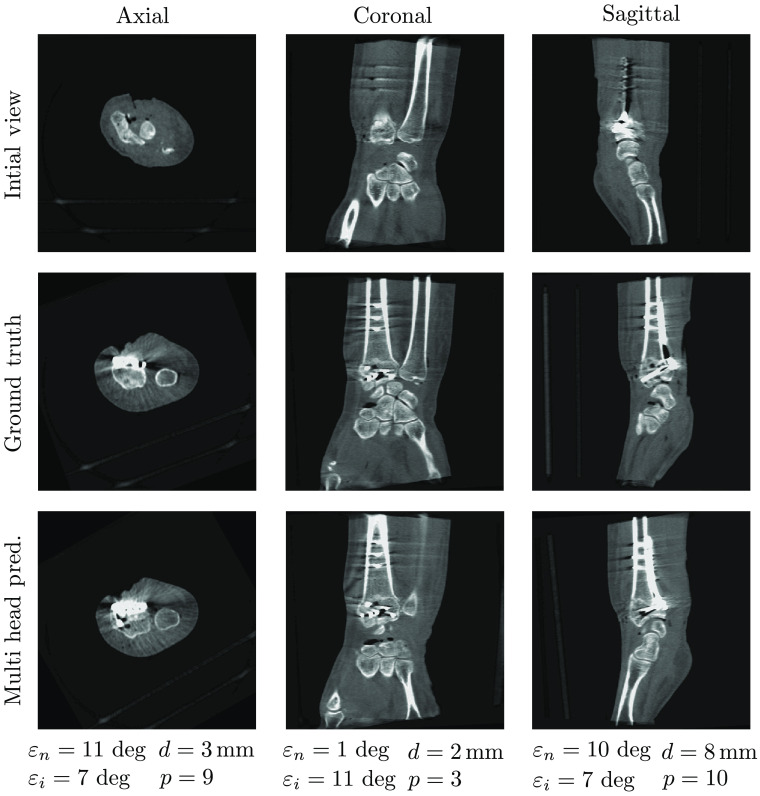
Example of automatic plane regression results by the multihead network for the clinical wrist data set.

**Fig. 6:**
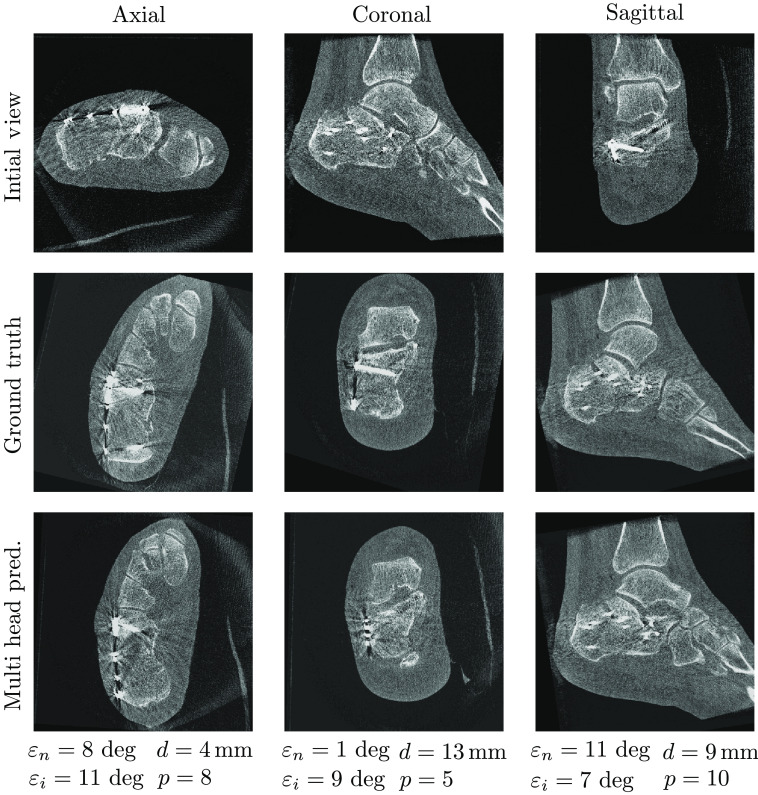
Example of automatic plane regression results by the multihead network for the clinical calcaneus data set.

**Fig. 7:**
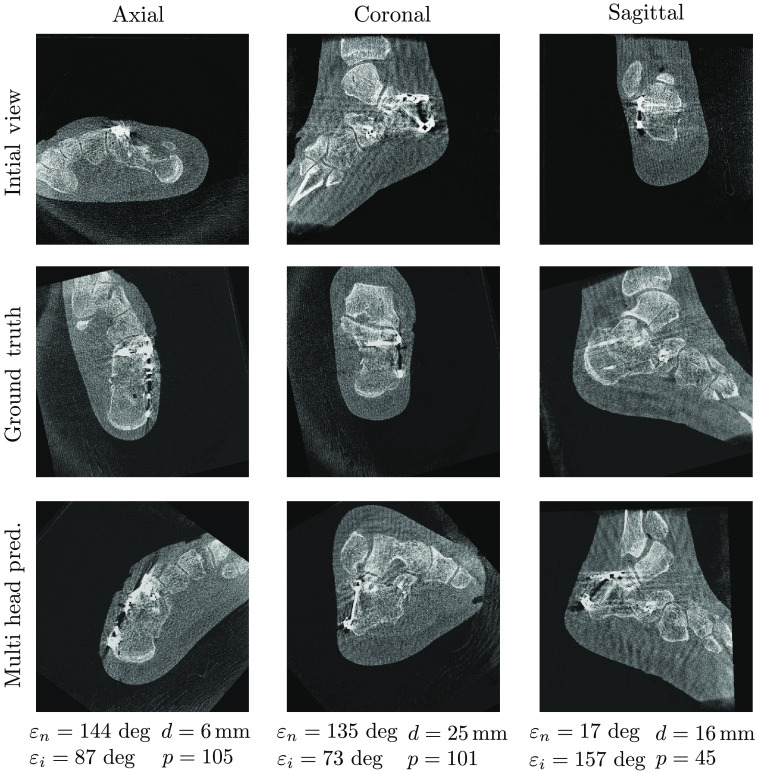
Failure example of the automatic plane regression by the multihead network for the clinical calcaneus data set.

Figures 5–7 show samples of the central planes of clinically acquired CBCT volumes and compare them with the manually adjusted standard planes and the automatically inferred predictions by the multihead network. For some cases, the algorithm was able to correct for in-plane rotation by 180 deg (Fig. 5) or for plane flips (Fig. 6). In contrast, Fig. 7 shows an error case in which the axial plane was predicted with a rotational offset of ∼90  deg, resulting in large deviations from the target standard planes.

## 5 Discussion and Conclusion

In this paper, we investigate the regression of standard planes for four different body regions. The volumes for which the standard planes should be regressed are acquired with mobile C-arm devices and therefore have a limited field of view. Furthermore, there is no standardized relationship between the C-arm device and the body region of interest, which means that the representation of the body region in the acquired volumes is not consistent. This also applies to the position of the body region in relation to the operating table. The target body regions are also in close proximity to flexible joints, such as knee, wrist, or ankle, leading to great variability of the input data and thus to considerably higher task complexity.

Despite this complex setting, our proposed method yields encouraging results with low median errors for the regressed angles and positions. The task of regressing the planes parameters can be performed equally well for orthogonal and oblique planes. Although axial planes are typically well regressed, the overall score is deteriorated by the coronal and sagittal planes. The normals of these planes are typically not as well defined, and small rotations by a few degrees are hardly noticed, even for a well-trained eye.

The experimental results reveal that the single-task networks already achieve very good accuracy. The ablation study on the required amount of data shows that, by adding further data to the training, we still obtain improvements in the achieved scores. Thus, we face the problem of generalization in three of four body regions.

Because the single-head MTL approach cannot improve over the single-task performance, we argue that a single head does not provide the required model complexity needed to learn an appropriate representation of data and task distribution. These shortcomings could be addressed by performing feature abstraction and combination in smaller consecutive steps, for example, by adding intermediate fully connected layers. This reasoning is supported by the observation that only the additional task-specific parameters of the multihead approach reduced the positioning and angulation errors of the standard planes.

The performance increase of the multihead approach mainly comes from an increase in position accuracy for all body regions—only slightly for the wrist but remarkable for the other regions. For this subtask, the pursuit for more robust estimates using feature sharing was successful: the position of the MPR planes at or near a joint gap for all body regions is likely to benefit from the robust features. For the direction estimation, the shared features provided a benefit only for the ankle. Especially for the wrist, for which the sizes of the relevant structures are smaller compared with the lower extremities, but also for the calcaneus, where the direction is not defined by the axis of a long bone, using a shared feature set has led to even higher angulation errors.

In the case that a larger amount of data is available, we see further potential to reduce the error for all network architectures. Then, no substantial differences between the analyzed architecture variants are to be expected. However, the MTL approach will help reduce the number of stored parameters and facilitate a common network for standard plane regression. Also, the network parameters need not be loaded depending on the scanned body part, which saves time during the execution.

The results show that good angle regression performance is obtained when the volumes are acquired with the body aligned to the imaging system axes as well as when the anatomy moderately deviates from the standard positioning. However, it fails in cases of severe deviations, such as when the body is rotated by more than 90 deg. For these cases, the applied augmentation pipeline does not help. The augmentation did not cover flips in the y direction because, in clinical practice, an upside-down flip of a wrist comes with a modification of configuration. In the case of the upper ankle or calcaneus, the upper ankle joint gets stretched more. Thus, applying the augmentation does not lead to clinically relevant data sets. Because at the present stage additional clinical data are not available and their clinical acquisition is seldom, more cadaver data are needed to sufficiently represent those poses. This also means that the results presented in this work do not show the full potential of this approach.

Nevertheless, the presented results with a mean error of the normal’s regression being 7.3 deg and mean error of the plane position being 6.1 mm meet the reported interrater variance in similar complex body regions of up to 6.3 deg for the normals and up to 9.3 mm for the plane position.^12^

Kausch et al.^12^ showed that human performance at adjusting the planes highly depends on the target region. In regions with well-defined landmarks and few anatomical variations, the plane adjustment’s interrater variance is low. In regions with well-defined landmarks and few anatomical variations, the plane adjustment’s interrater variance is low. However, this variance is substantially higher in regions for which less reliable landmarks can be identified. For the presented anatomies, no such variance estimates are available yet. This limits the interpretability of our results because no well-defined reference values for clinically required precision can serve as a standard. Although such a comparative analysis should be addressed in follow-up studies, we generally see promising results of our proposed method that fit well within the error bounds of related studies of anatomies with comparable complexity.^12^

A benefit of the direct standard plane parameter regression is the reduced amount of annotation data per data set. Costly annotations of landmarks or even segmentation of bones can be omitted and are replaced by comparably cheap adjustments of the standard planes. Also, the implementation of specific rules per body region to obtain the parameters of the landmarks is omitted. Thus, the direct MPR plane parameter regression provides a generic tool for plane parameter estimation: it requires cheap training labels, and it integrates well into the surgical workflow through a fast adjustment of the planes during loading of the volume.

## 6 Appendix A. Postprocessing of Regressed Values

In this section, the influence of the postprocessing of the regressed angles is evaluated. For that, $\epsilon_{n}$ and $\epsilon_{i}$ are calculated with and without postprocessing and their values are compared. The analysis of the influence on the postprocessing to the single parts of the score for $6D_{xy}$ representation (Table 5) shows that the postprocessing helps to improve $\epsilon_{n}$ as well as $\epsilon_{i}$ by up to 1.89 deg. As the translation remains untouched by the postprocessing, the translation error d does not change.

**Table 5:** Comparison of the errors directly obtained by the network (regressed) and after postprocessing ensuring orthogonality of respective planes (postproc.) using the $6D_{xy}$ rotation representation.

	$\varepsilon_{n}$ (deg)	$\varepsilon_{i}$ (deg)	Score
Calcaneus			
Regressed	8.77 ± 0.60	8.34 ± 0.44	8.92 ± 0.51
Postproc.	**8.08 ± 0.38**	**8.09 ± 0.45**	**8.46 ± 0.63**
Ankle			
Regressed	7.11 ± 0.48	6.58 ± 0.29	6.70 ± 0.35
Postproc.	**6.61 ± 0.34**	**6.37 ± 0.31**	**6.32 ± 0.25**
Knee			
Regressed	8.60 ± 0.98	8.45 ± 0.63	8.22 ± 0.71
Postproc.	**6.71 ± 0.63**	**7.07 ± 0.95**	**6.80 ± 0.55**
Wrist			
Regressed	8.76 ± 1.19	8.84 ± 0.94	8.48 ± 1.09
Postproc.	**7.74 ± 1.14**	**8.72 ± 0.64**	**7.85 ± 0.94**

Note: Bold values represent the lowest values of error class for each body region.

## 7 Appendix B. Hyperparameter Optimization

For hyperparameter optimization, one fold was used, and individual hyperparameter optimizations were performed for the different rotation descriptions in the baseline network. The parameter space was sampled randomly. In Table 6, the search space for each hyperparameter evaluated as well as the sampling value for the $6D_{xy}$ representation are listed. This method results in an offset of typically 0.1 and maximum 0.4 score points.

**Table 6:** Search space hyperparameters, sampling distribution, and best configuration for the plane regression task as result of random search hyperparameter optimization.

Hyperparameter	Sampling distribution	Sampling value
Learning rate	s∼log U(0.0001,0.01)	0.00164
Learning rate decay	s∼log U(0.2,0.9)	0.27291
Learning rate decay step	s∼U(20,80)	75
Momentum	s∼log U(0.5,0.99)	0.957437
Batch size	s∼U(5,12)	9

## 8 Appendix C. Detailed Results

In this appendix, the detailed results of the evaluation of data ablation can be found. Table 7 summarizes the results of evaluation of data ablation in standard plane regression of calcaneus, ankle, knee, and wrist body regions.

**Table 7:** Summarized results of evaluation of data ablation in standard plane regression of calcaneus, ankle, knee, and wrist body regions.

	d (mm)	$\varepsilon_{n}$ (deg)	$\varepsilon_{i}$ (deg)	Score
Calcaneus				
100%	9.94 ± 1.92	8.08 ± 0.38	8.09 ± 0.45	8.46 ± 0.63
80%	9.96 ± 1.43	9.16 ± 1.13	8.99 ± 0.67	9.29 ± 1.05
60%	11.20 ± 2.02	9.36 ± 1.19	9.22 ± 0.34	9.70 ± 0.75
40%	15.45 ± 7.49	10.95 ± 0.64	11.41 ± 1.27	11.94 ± 1.73
Ankle				
100%	5.43 ± 0.25	6.61 ± 0.34	6.37 ± 0.31	6.32 ± 0.25
80%	5.44 ± 0.27	6.34 ± 0.45	6.36 ± 0.42	6.17 ± 0.34
60%	6.59 ± 0.33	7.11 ± 0.99	6.88 ± 0.48	6.96 ± 0.67
40%	7.72 ± 1.28	9.16 ± 1.61	9.79 ± 2.21	9.00 ± 1.59
Knee				
100%	6.81 ± 0.47	6.71 ± 0.63	7.07 ± 0.95	6.80 ± 0.55
80%	7.24 ± 0.72	7.57 ± 0.77	7.55 ± 0.97	7.50 ± 0.73
60%	10.18 ± 2.27	8.89 ± 0.89	9.25 ± 0.73	9.22 ± 0.73
40%	10.13 ± 1.40	10.22 ± 1.21	10.30 ± 1.31	10.22 ± 1.25
Wrist				
100%	7.27 ± 1.08	7.74 ± 1.14	8.72 ± 0.64	7.85 ± 0.94
80%	8.63 ± 1.75	8.88 ± 1.18	9.65 ± 0.83	8.98 ± 1.01
60%	8.29 ± 1.07	9.14 ± 0.73	10.49 ± 1.46	9.24 ± 0.81
40%	9.14 ± 1.04	11.98 ± 1.64	12.53 ± 1.31	11.52 ± 1.33
